# Cumulative burden of metabolic syndrome and its components on the risk of atrial fibrillation: a nationwide population-based study

**DOI:** 10.1186/s12933-021-01215-8

**Published:** 2021-01-19

**Authors:** Hyo-Jeong Ahn, Kyung-Do Han, Eue-Keun Choi, Jin-Hyung Jung, Soonil Kwon, So-Ryoung Lee, Seil Oh, Gregory Y. H. Lip

**Affiliations:** 1 Department of Internal Medicine, Seoul National University Hospital, 101 Daehak-ro, Jongno-gu, Seoul, 03080 Republic of Korea; 2 Department of Statistics and Actuarial Science, Soongsil University, Seoul, Republic of Korea; 3 Department of Internal Medicine, Seoul National University College of Medicine, Seoul, Republic of Korea; 4 Department of Medical Statistics, College of Medicine, The Catholic University of Korea, Seoul, Republic of Korea; 5 Liverpool Centre for Cardiovascular Science, University of Liverpool and Liverpool Chest & Heart Hospital, Liverpool, UK; 6 Department of Clinical Medicine, Aalborg University, Aalborg, Denmark

**Keywords:** Metabolic syndrome, Metabolic burden, Atrial fibrillation

## Abstract

**Background:**

The metabolic syndrome (MetS) and its components are associated with the development of atrial fibrillation (AF). However, the impact of time-burden of MetS on the risk of AF is unknown. We investigated the effect of the cumulative longitudinal burden of MetS on the development of AF.

**Methods:**

We included 2 885 189 individuals without AF who underwent four annual health examinations during 2009–2013 from the database of the Korean national health insurance service. Metabolic burdens were evaluated in the following three ways: (1) cumulative number of MetS diagnosed at each health examination (0–4 times); (2) cumulative number of each MetS component diagnosed at each health examination (0–4 times per MetS component); and (3) cumulative number of total MetS components diagnosed at each health examination (0 to a maximum of 20). The risk of AF according to the metabolic burden was estimated using Cox proportional-hazards models.

**Results:**

Of all individuals, 62.4%, 14.8%, 8.7%, 6.5%, and 7.6% met the MetS diagnostic criteria 0, 1, 2, 3, and 4 times, respectively. During a mean follow-up of 5.3 years, the risk of AF showed a positive association with the cumulative number of MetS diagnosed over four health examinations: adjusted hazard ratios (HRs) with 95% confidence intervals (CIs) of 1, 2, 3, and 4 times compared to 0 times were 1.18 (1.13–1.24), 1.31 (1.25–1.39), 1.46 (1.38–1.55), and 1.72 (1.63–1.82), respectively; *P* for trend < 0.001. All five components of MetS, when diagnosed repeatedly, were independently associated with an increased risk of AF: adjusted HR (95% CI) from 1.22 (1.15–1.29) for impaired fasting glucose to 1.96 (1.87–2.07) for elevated blood pressure. As metabolic components were accumulated from 0 to 20 counts, the risk of AF also gradually increased up to 3.1-fold (adjusted HR 3.11, 95% CI 2.52–3.83 in those with 20 cumulative components of MetS), however, recovery from MetS was linked to a decreased risk of AF.

**Conclusions:**

Given the positive correlations between the cumulative metabolic burdens and the risk of incident AF, maximal effort to detect and correct metabolic derangements even before MetS development might be important to prevent AF and related cardiovascular diseases.

## Background

Atrial fibrillation (AF) is the most common sustained cardiac arrhythmia associated with an increased risk of stroke, heart failure, myocardial infarction (MI), dementia, and death [1–3]. The prevalence of AF in adults is 2%-4%, and the lifetime AF risk was recently estimated as 1 in 3 individuals of European ancestry at an index age of 55 years [4, 5]. As the incidence of AF is expected to increase, management of AF has been emphasized as integrating stroke prevention, symptom control, risk factors management, and lifestyle modification [6, 7].

Metabolic syndrome (MetS) is a cluster of metabolic disorders including glucose intolerance, low level of high-density lipoprotein cholesterol (HDL-C), high level of triglyceride (TG), obesity, and hypertension [8, 9]. MetS, frequently combined with other atherosclerotic risk factors, has become a pandemic that increases the risk of cardiovascular morbidity and mortality [8, 10].

As both AF and MetS lead to significant cardiovascular diseases, imposing an enormous healthcare burden, many studies have attempted to reveal the relationship between MetS and AF development; MetS and its components are known to be associated with AF development [11, 12]. Elevated levels of inflammation, oxidative stress, and mechanical stimuli to the atrium have been proposed as the underlying pathophysiology of AF development in MetS [10, 13, 14]. Also, insulin resistance has been identified as one of the common physiologic manifestations significantly associated with both AF and MetS [15–18]. Notably, Type 2 diabetes is a well-known independent risk factor of AF, and one of the components of CHA_2_DS_2_-VASc-score used to identify high-risk AF patients for stroke [19, 20]. However, MetS status and its components are variable as they can change over time dynamically. A recent study demonstrated the association between the exposure duration or extent of metabolic disturbances and the increased risk of MI and stroke, which are AF-related complications [21]. Nonetheless, to what extent the time-burden of MetS affects the risk of AF itself has not been evaluated. Therefore, we aimed to investigate the effect of the cumulative longitudinal burden of MetS on AF development through a nationwide population-based cohort analysis.

## Methods

We defined a population-based cohort from the National Health Information Database (NHID) incorporating all data from the National Health Insurance Service, which covers the entire population of the Republic of Korea (hereafter, Korea). All insured adults aged 40 years and older are recommended to undergo biannual general health screening without cost [22]. Not only the regular health examination records, including laboratory results, anthropometric measurements, and detailed lifestyle questionnaires, but also sociodemographic data, income-based insurance contributions, prescription records, inpatient and outpatient usage, and date of death of all insured Koreans are available in the NHID [22, 23]. The Institutional Review Board at the Seoul National University Hospital (E-2009–091-1157) authorized this study.

We selected 3 981 515 adults aged ≥ 20 years who underwent four serial health examinations between January 1, 2009, and December 31, 2013, from the NHID. Individuals with a prior history of AF and missing values of health examination data or covariates were excluded (Additional file 1: Fig. S1). Finally, 2 885 189 adults were included in the analysis.

### Evaluation of metabolic syndrome and the influence of cumulative metabolic burden

MetS was defined using modified waist circumference (WC) criteria of the Korean Society for the Study of Obesity and the guidelines of the National Cholesterol Education Program Third Adult Treatment Panel (NCEP-ATP III) as the presence of ≥ 3 of the following: increased WC [≥ 90 cm in men or ≥ 85 cm in women], elevated TG [≥ 150 mg/dL (1.7 mmol/L) or drug treatment for elevated TG], low HDL-C [< 40 mg/dL (1 mmol/L) in men and < 50 mg/dL (1.3 mmol/L) in women or drug treatment for low HDL-C], elevated blood pressure [systolic blood pressure ≥ 130 mmHg or diastolic blood pressure ≥ 85 mmHg or current use of antihypertensives], and impaired fasting glucose [fasting plasma glucose ≥ 100 mg/dL (5.6 mmol/L) or current use of antidiabetics] [8, 9, 24].

**Fig. 1:**
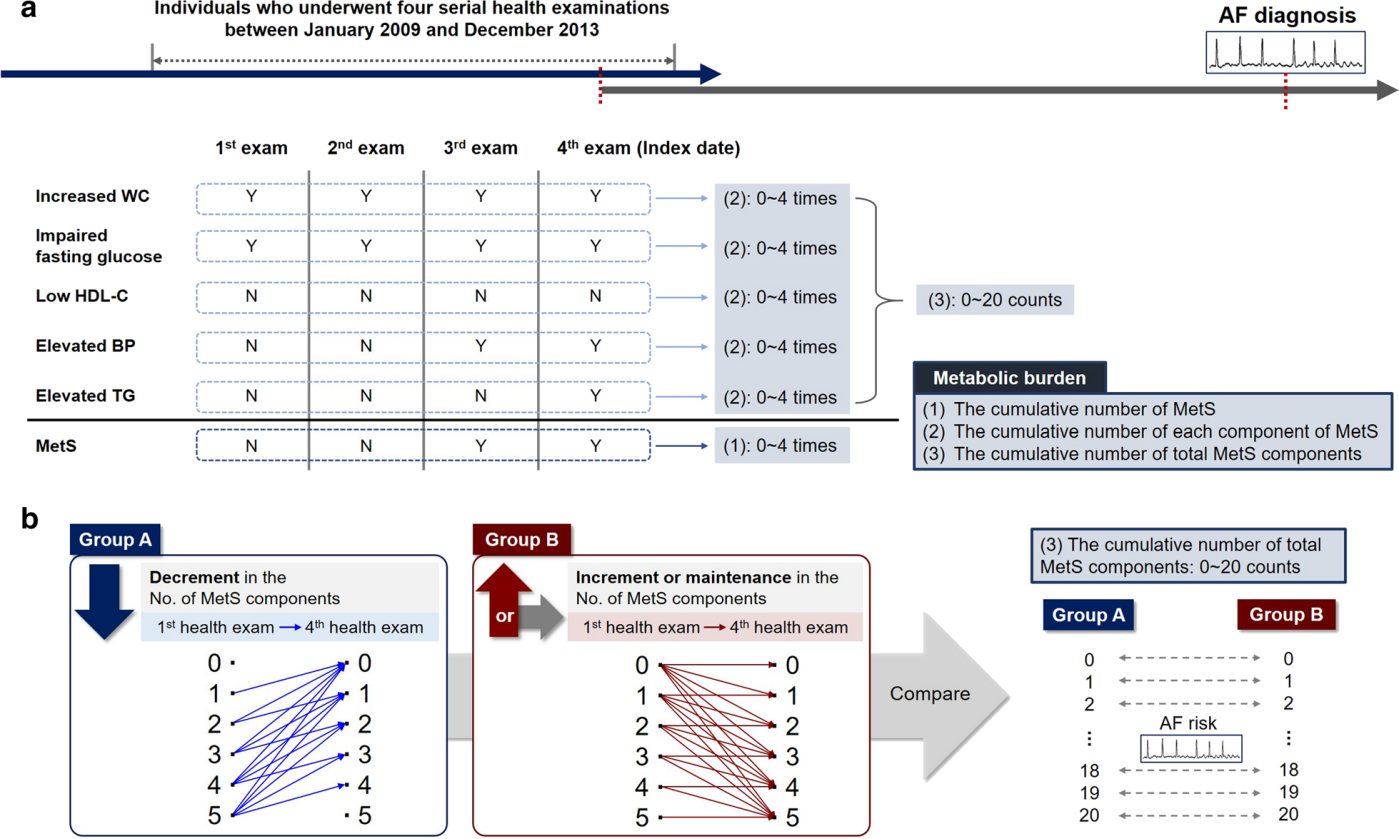
**a** Overall scheme of study and three different ways of defining metabolic burdens. **b** Evaluation of temporal trends in the metabolic burden on the incidence of AF. *AF* atrial fibrillation, *MetS* metabolic syndrome, *WC* waist circumference, *HDL-C* high-density lipoprotein cholesterol, *BP* blood pressure, *TG* triglycerides, *Y* yes, *N *no

At each health examination, the presence of MetS itself and the number of fulfilled components of MetS were calculated. “Metabolic burden” is defined as the following three multifaceted ways during four health examinations: (1) cumulative number of MetS diagnosed at each health examination (0–4 times); (2) cumulative number of each MetS component diagnosed at each health examination (0–4 times per each MetS component); and (3) cumulative number of total MetS components diagnosed at each health examination (0 to a maximum of 20). The overall scheme of the study and the three different ways of defining metabolic burdens are shown in Fig. 1a.

To estimate the influence of temporal trends in metabolic burden on AF incidence, we compared the number of metabolic components at the first health examination with that at the last examination. The total study population was divided into two groups according to the change in the number of metabolic components (Fig. 1b).

Group A was defined as the group with a decrease in the number of metabolic components, whereas group B was defined as that with an increase or maintenance in the number of metabolic components at the last health examination compared to that in the first examination. We stratified groups A and B according to the total number of MetS components diagnosed during the four health examinations. At each cumulative number of total MetS components, we matched two groups and compared the risk of AF (hazard ratio [HR] with 95% confidence interval [CI] of group A was enumerated with reference to Group B).

### Covariates, follow-up, and clinical outcomes

Baseline characteristics of the participants were designated as the data of the last health examination (index date), which includes sociodemographic data, income-based insurance contributions, laboratory results, anthropometric measurements, comorbidities (diabetes mellitus, hypertension, dyslipidemia, peripheral artery disease, chronic obstructive pulmonary disease, myocardial infarction, heart failure, cancer, and chronic kidney disease) and answers to detailed lifestyle questionnaires. We investigated the risk of new-onset AF using the International Classification of Diseases, Tenth Revision (ICD-10), codes with inpatient and outpatient records. Detailed definitions of AF and comorbidities are summarized in Additional file 1: Table S1 [22]. The follow-up period was the time from the index date to the occurrence of AF or December 31, 2018, whichever came first.

### Statistical analysis

Data are reported as means ± standard deviations for continuous variables and number (%) for categorical variables. To evaluate significant differences in baseline characteristics among groups categorized by the number of MetS components, one-way analysis of variance and chi-square test were used. The incidence rate of AF was computed by dividing new-onset AF cases by the total follow-up duration and presented as per 1000 person-years (PY). The association between the frequency of MetS status and the incidence of AF was estimated using Cox proportional hazards regression models. The risk of AF according to the number of MetS compared to the non-MetS group was expressed as HRs with 95% CIs. Model 1 represents an unadjusted risk, and Model 2 was adjusted for age, sex, smoking status (never smoker, ex-smoker, or current smoker), alcohol intake (non, mild, or heavy drinker, g/day), regular exercise (performing > 30 min of moderate physical activity ≥ 5 times a week or > 20 min of vigorous physical activity ≥ 3 times a week), and low-income level (income in lower 20% among the entire Korean population of subjects supported by the medical aid program) [25]. Model 3 was additionally adjusted for WC, systolic blood pressure, fasting glucose, logarithm of TG, and HDL-C levels.

To verify the robustness of our results, we performed several subgroups and sensitivity analyses with regard to the association between the cumulative number of total MetS components diagnosed at each health examination (0 to a maximum of 20) and the risk of AF. First, we investigated the association between the cumulative frequency of metabolic components and the risk of AF according to sex and the presence of obesity to determine potential effect modifications. We also conducted an analysis by excluding participants with AF diagnosed in the first 2 years of follow-up to minimize the effect of reverse causality. Data collection and statistical analyses were performed using SAS version 9.4 (SAS Institute, Cary, NC).

## Results

The baseline characteristics of 2 885 189 participants grouped according to the number of MetS diagnosed over four health examinations are summarized in Table 1. The mean participant age was 44.5 ± 10.9 years, and 2 058 645 (71.4%) were men. Individuals were divided into five groups: 1 800 268 (62.4%), 428 143 (14.8%), 250 073 (8.7%), 188 847 (6.5%), and 217 858 (7.6%) who met the diagnostic criteria of MetS 0, 1, 2, 3, and 4 times, respectively. Diabetes, hypertension, and dyslipidemia were present in 7.1%, 20.7%, and 17.3% of the total population, respectively. The prevalence of comorbidities was higher as individuals fulfilled more MetS criteria.

**Table 1:** Baseline characteristics of the total study population according to the cumulative number of MetS diagnosed at each health examination (0 to 4 times)

	Total	The number of the presence of metabolic syndrome					p
		0	1	2	3	4	
No. of participants (%)	2,885,189 (100.0)	1,800,268 (62.4)	428,143 (14.8)	250,073 (8.7)	188,847 (6.5)	217,858 (7.6)	
Age (years)	44.54 ± 10.85	42.38 ± 10.39	46.30 ± 10.58	47.93 ± 10.66	49.11 ± 10.49	51.07 ± 10.16	< 0.001
Sex							
Male	2,058,645 (71.4)	1,197,934 (66.5)	337,756 (78.9)	200,399 (80.1)	152,483 (80.7)	170,073 (78.1)	< 0.001
Female	826,544 (28.7)	602,334 (33.5)	90,387 (21.1)	49,674 (19.9)	36,364 (19.3)	47,785 (21.9)	
Smoking							< 0.001
Never smoker	1,369,623 (47.5)	938,078 (52.1)	174,286 (40.7)	97,977 (39.2)	72,215 (38.2)	87,067 (40.0)	
Ex-smoker	601,896 (20.9)	336,101 (18.7)	100,158 (23.4)	61,532 (24.6)	48,473 (25.7)	55,632 (25.5)	
Current smoker	913,670 (31.7)	526,089 (29.2)	153,699 (35.9)	90,564 (36.2)	68,159 (36.1)	75,159 (34.5)	
Alcohol consumption^a^							< 0.001
Non-drinker	1,131,332 (39.2)	728,676 (40.5)	156,540 (36.6)	91,741 (36.7)	69,234 (36.7)	85,141 (39.1)	
Mild to moderate drinker	1,511,675 (52.4)	947,766 (52.7)	228,079 (53.3)	130,842 (52.3)	97,706 (51.7)	107,282 (49.2)	
Heavy drinker	242,182 (8.4)	123,826 (6.9)	43,524 (10.2)	27,490 (11.0)	21,907 (11.6)	25,435 (11.7)	
Regular exercise^b^	642,402 (22.3)	390,171 (21.7)	98,815 (23.1)	57,963 (23.2)	43,981 (23.3)	51,472 (23.6)	< 0.001
Low income^c^	579,366 (20.1)	329,917 (18.3)	89,519 (20.9)	55,953 (22.4)	43,219 (22.9)	60,758 (27.9)	< 0.001
Comorbidities							
Diabetes mellitus	204,495 (7.1)	35,151 (2.0)	28,569 (6.7)	28,964 (11.6)	35,968 (19.1)	75,843 (34.8)	< 0.001
Hypertension	597,210 (20.7)	173,667 (9.7)	106,689 (24.9)	89,778 (35.9)	88,335 (46.8)	138,741 (63.7)	< 0.001
Dyslipidemia	499,947 (17.3)	177,145 (9.8)	87,677 (20.5)	68,089 (27.2)	64,119 (34.0)	102,917 (47.2)	< 0.001
PAD	198,421 (6.9)	76,179 (4.2)	32,740 (7.7)	25,468 (10.2)	24,067 (12.7)	39,967 (18.3)	< 0.001
COPD	590,393 (20.5)	344,502 (19.1)	89,663 (20.9)	56,012 (22.4)	44,029 (23.3)	56,187 (25.8)	< 0.001
MI	37,979 (1.3)	15,751 (0.9)	6212 (1.5)	4545 (1.8)	4269 (2.3)	7202 (3.3)	< 0.001
HF	32,611 (1.13)	12,590 (0.7)	5126 (1.2)	4122 (1.65)	3952 (2.09)	6821 (3.13)	< 0.001
Cancer	42,378 (1.5)	24,148 (1.3)	6579 (1.5)	4077 (1.6)	3364 (1.8)	4210 (1.9)	< 0.001
CKD	56,830 (2.0)	23,580 (1.3)	8867 (2.1)	7108 (2.8)	6702 (3.6)	10,573 (4.9)	< 0.001
Laboratory findings							
BMI (kg/m^2^)	23.84 ± 3.16	22.72 ± 2.66	24.80 ± 2.75	25.68 ± 2.88	26.34 ± 3.00	26.91 ± 3.22	< 0.001
Waist circumference (cm)	80.79 ± 8.85	77.55 ± 7.76	83.72 ± 7.25	86.15 ± 7.40	87.96 ± 7.58	89.50 ± 8.17	< 0.001
Systolic BP (mmHg)	121.68 ± 13.55	118.11 ± 12.45	125.18 ± 12.66	127.71 ± 12.91	129.38 ± 13.17	130.65 ± 13.81	< 0.001
Diastolic BP (mmHg)	76.59 ± 9.48	74.39 ± 8.84	78.80 ± 8.97	80.37 ± 9.19	81.39 ± 9.44	81.94 ± 9.85	< 0.001
Fasting glucose (mg/dL)	96.7 ± 20.65	91.64 ± 13.28	98.76 ± 19.15	103.09 ± 23.41	108.24 ± 28.56	117.16 ± 36.52	< 0.001
Total cholesterol (mg/dL)	195.09 ± 35.07	191.10 ± 32.91	200.74 ± 35.66	203.00 ± 37.03	203.23 ± 38.35	200.80 ± 40.45	< 0.001
HDL cholesterol (mg/dL)	54.78 ± 14.83	58.13 ± 14.4	51.13 ± 13.84	49.00 ± 13.74	47.55 ± 13.41	47.08 ± 13.73	< 0.001
LDL cholesterol (mg/dL)	113.98 ± 32.14	111.91 ± 30.09	118.58 ± 33.16	118.82 ± 34.72	117.50 ± 35.94	113.46 ± 37.65	< 0.001
eGFR (ml/min/1.73m2)	92.75 ± 37.86	94.19 ± 38.14	91.57 ± 37.51	90.37 ± 37.02	89.73 ± 38.14	88.56 ± 36.24	< 0.001
Triglyceride (mg/dL)	135.98 ± 92.48	106.77 ± 64.50	160.86 ± 94.11	184.62 ± 106.67	201.99 ± 114.90	215.47 ± 126.08	< 0.001
Medication							
Statin	245,676 (8.5)	56,971 (3.2)	39,209 (9.2)	36,314 (14.5)	38,637 (20.5)	74,545 (34.2)	< 0.001

Data are presented as means ± SD or No. (Percentages)

Percentages may not total 100 because of rounding

*MetS *metabolic syndrome, *PAD* peripheral artery disease, *COPD* chronic obstructive pulmonary disease, *MI* myocardial infarction, *HF* heart failure, *CKD* chronic kidney disease, *BMI* body mass index, *BP* blood pressure, *HDL* high-density lipoprotein, *LDL* low-density lipoprotein, *eGFR* estimated glomerular filtration rate

^a^Alcohol consumption denotes as following

Non-drinker: alcohol consumption 0 g

Mild to moderate drinker: alcohol consumption > 0 g to < 30 g per day

Heavy drinker: alcohol consumption ≥ 30 g per day

^b^Regular exercise denotes performing > 30 min of moderate-intensity exercise (e.g. brisk pace walking, tennis doubles, or bicycling leisurely) ≥ 5 times a week or > 20 min of vigorous-intensity exercise (e.g. running, climbing, fast cycling, or aerobics) ≥ 3 times a week

^c^Low income denotes income belongs to lower 20% among entire Korean population of subjects supported by the Medical Aid program

### Accumulation of metabolic burden and the risk of AF

**Fig. 2:**
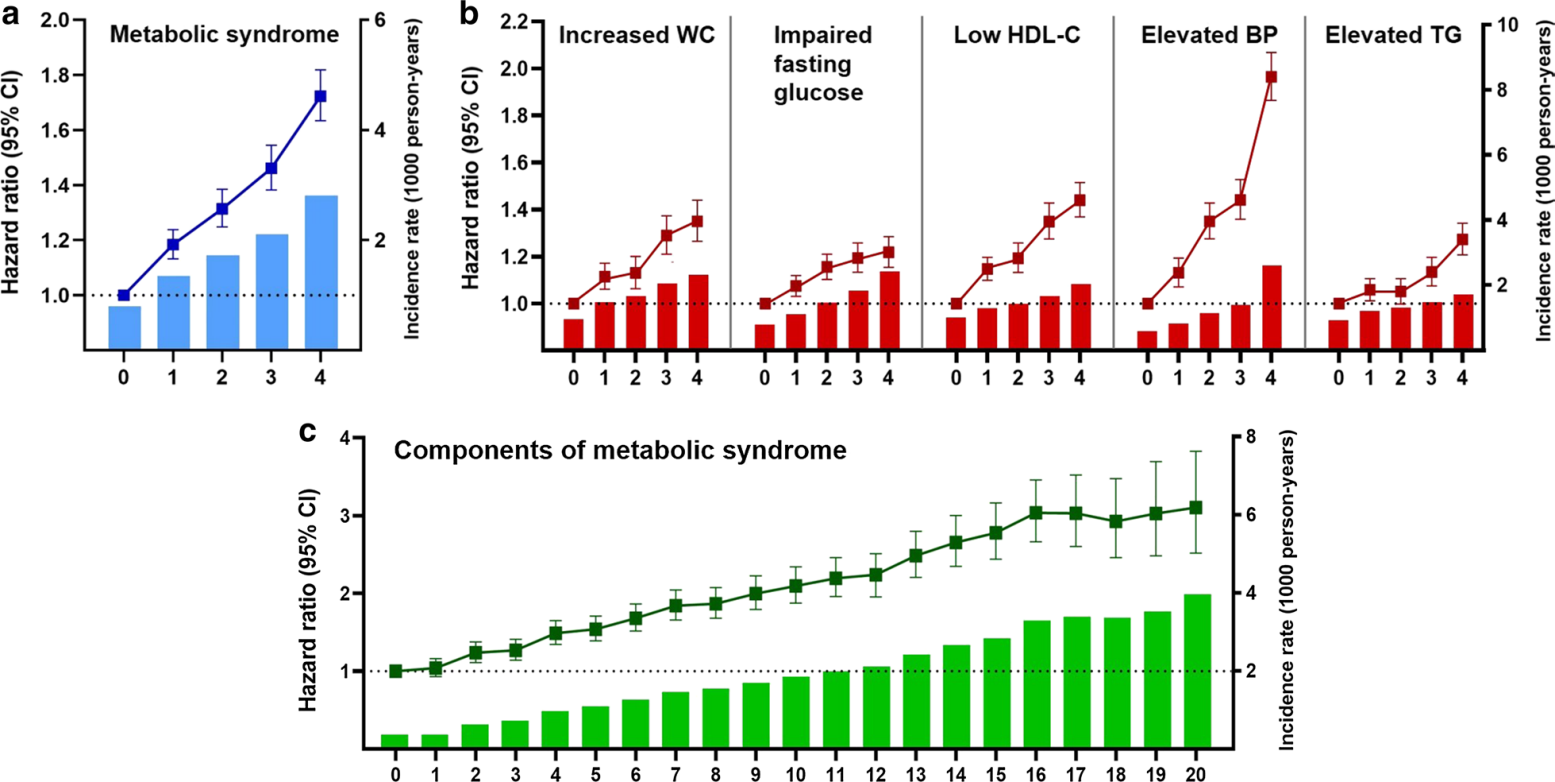
Incidence of atrial fibrillation according to the **a** cumulative number of MetS diagnosed at each health examination, **b** cumulative number of each component of MetS diagnosed at each health examination, and **c** cumulative number of total MetS components diagnosed during four health examinations. *WC* waist circumference, *HDL-C* high-density lipoprotein cholesterol, *TG* triglycerides

The follow-up period was 5.3 ± 0.3 years. The incidence of AF, according to the number of MetS and the number of MetS components during the four health examinations are described in Table 2. The risk of AF showed a positive association with the cumulative number of MetS diagnosed at each health examination: adjusted HRs with 95% CIs of groups meeting the diagnostic criteria of MetS 1, 2, 3, and 4 times compared to 0 time were 1.18 (1.13–1.24), 1.31 (1.25–1.39), 1.46 (1.38–1.55), and 1.72 (1.63–1.82), *P* for trend < 0.001. The association is illustrated in Fig. 2a.

**Table 2:** The risk of atrial fibrillation according to the cumulative number of MetS and each component diagnosed during four health examinations (0 to 4 times)

The number of meeting the component	No. of participants	AF	IR (1000PY)	HR (95% CI)		
				Model 1	Model 2	Model 3
Metabolic syndrome						
0	1,800,268	7584	0.80	1.00 (Reference)	1.00 (Reference)	1.00 (Reference)
1	428,143	3025	1.35	1.69 (1.62–1.77)	1.21 (1.16–1.27)	1.18 (1.13–1.24)
2	250,073	2248	1.72	2.16 (2.06–2.27)	1.37 (1.31–1.44)	1.31 (1.25–1.39)
3	188,847	2071	2.10	2.64 (2.52–2.77)	1.55 (1.48–1.63)	1.46 (1.38–1.55)
4	217,858	3184	2.81	3.54 (3.39–3.69)	1.87 (1.80–1.95)	1.72 (1.63–1.82)
				p < 0.001	p < 0.001	p < 0.001
Increased waist circumference						
0	2,063,537	10,304	0.95	1.00 (Reference)	1.00 (Reference)	1.00 (Reference)
1	298,977	2292	1.47	1.55 (1.48–1.63)	1.24 (1.19–1.30)	1.12 (1.06–1.17)
2	171,305	1487	1.66	1.76 (1.67–1.86)	1.31 (1.24–1.39)	1.13 (1.06–1.20)
3	147,806	1570	2.04	2.16 (2.05–2.28)	1.56 (1.48–1.64)	1.29 (1.21–1.37)
4	203,564	2459	2.32	2.46 (2.35–2.57)	1.76 (1.69–1.84)	1.35 (1.27–1.44)
				p < 0.001	p < 0.001	p < 0.001
Impaired fasting glucose						
0	1,290,283	5344	0.78	1.00 (Reference)	1.00 (Reference)	1.00 (Reference)
1	682,047	3952	1.10	1.41 (1.35–1.47)	1.11 (1.06–1.16)	1.08 (1.03–1.12)
2	385,692	2949	1.46	1.87 (1.79–1.95)	1.22 (1.17–1.28)	1.16 (1.10–1.21)
3	244,101	2323	1.82	2.33 (2.22–2.45)	1.30 (1.23–1.36)	1.19 (1.13–1.26)
4	283,066	3544	2.41	3.08 (2.96–3.22)	1.39 (1.33–1.45)	1.22 (1.15–1.29)
				p < 0.001	p < 0.001	p < 0.001
Low HDL-C						
0	1,817,377	9598	1.00	1.00 (Reference)	1.00 (Reference)	1.00 (Reference)
1	439,918	2973	1.29	1.29 (1.24–1.34)	1.17 (1.12–1.22)	1.15 (1.10–1.20)
2	242,845	1817	1.43	1.43 (1.36–1.50)	1.23 (1.17–1.29)	1.19 (1.13–1.26)
3	182,602	1585	1.65	1.65 (1.56–1.74)	1.40 (1.32–1.47)	1.35 (1.28–1.43)
4	202,447	2139	2.02	2.02 (1.92–2.11)	1.52 (1.45–1.59)	1.44 (1.37–1.52)
				p < 0.001	p < 0.001	p < 0.001
Elevated blood pressure						
0	994,829	3048	0.58	1.00 (Reference)	1.00 (Reference)	1.00 (Reference)
1	568,202	2461	0.82	1.42 (1.34–1.49)	1.12 (1.07–1.19)	1.13 (1.07–1.19)
2	417,544	2492	1.14	1.96 (1.86–2.07)	1.33 (1.26–1.41)	1.35 (1.28–1.43)
3	343,192	2510	1.40	2.41 (2.29–2.54)	1.41 (1.34–1.49)	1.44 (1.36–1.53)
4	561,422	7601	2.59	4.48 (4.29–4.67)	1.97 (1.88–2.05)	1.96 (1.87–2.07)
				p < 0.001	p < 0.001	p < 0.001
Elevated TG						
0	1,284,347	6255	0.92	1.00 (Reference)	1.00 (Reference)	1.00 (Reference)
1	506,800	3189	1.20	1.30 (1.24–1.35)	1.07 (1.02–1.11)	1.06 (1.01–1.11)
2	352,286	2415	1.31	1.42 (1.35–1.48)	1.06 (1.01–1.12)	1.05(1.00–1.11)
3	316,707	2435	1.46	1.59 (1.51–1.66)	1.15 (1.09–1.20)	1.13 (1.08–1.20)
4	425,049	3818	1.71	1.86 (1.79–1.94)	1.29 (1.24–1.34)	1.27 (1.21–1.34)
				p < 0.001	p < 0.001	p < 0.001

Model 1 is unadjusted

Model 2 is adjusted for age, sex, smoking status, alcohol intake, regular exercise, and low income

Model 3 is adjusted for age, sex, smoking status, alcohol intake, regular exercise, low income, waist circumference, systolic blood pressure, fasting glucose, logarithm of TG, and HDL-C level

*AF *atrial fibrillation, *IR* incidence rate, *PY* person-years, *HR* hazard ratio, *CI* confidence interval, *HDL-C* high-density lipoprotein cholesterol, *TG* triglycerides

All five MetS components were independently associated with the risk of AF. As the number of MetS components accumulated, the risk of AF also increased (Table 2 and Fig. 2b). The effect of each MetS component on the risk of AF was different. The adjusted HR (95% CI) of each MetS component, when diagnosed four times consecutively, was as follows: 1.96 (1.87–2.07) for elevated blood pressure, 1.44 (1.37–1.52) for low HDL-C, 1.35 (1.27–1.44) for increased WC, 1.27 (1.21–1.34) for elevated TG, and 1.22 (1.15–1.29) for impaired fasting glucose.

**Fig. 3:**
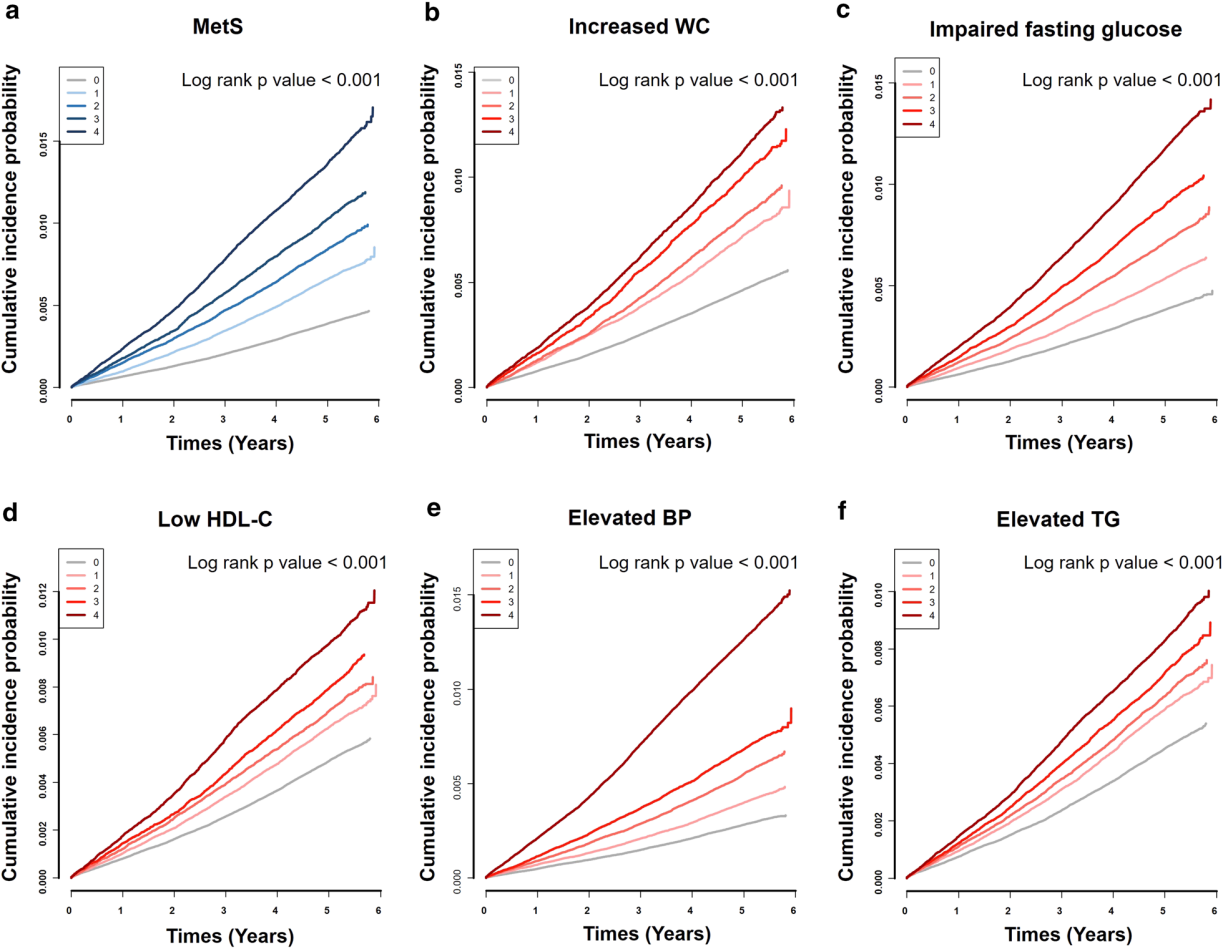
Kaplan‒Meier estimates of cumulative incidence probability of AF according to the cumulative number of **a** MetS, **b** increased waist circumference, **c** impaired fasting glucose, **d** low HDL-C, **e** elevated blood pressure, and **f** elevated TG during four health examinations. *AF* atrial fibrillation, *MetS* metabolic syndrome, *WC* waist circumference, *HDL-C* high-density lipoprotein cholesterol, *BP* blood pressure, *TG* triglycerides

Table 3 and Fig. 2c show the relationship between metabolic burden as the cumulative number of all MetS components during the four health examinations and the risk of AF. As participants had an accumulation of metabolic components from 0 to 20 counts, the risk of AF also gradually increased by approximately 3.1-fold compared to those without any metabolic components (HR 3.11, 95% CI 2.52–3.83 in those with 20 components of MetS). Kaplan–Meier estimates of the cumulative incidence probability of AF are shown in Fig. 3.

**Table 3:** The risk of atrial fibrillation according to the cumulative number of total MetS components diagnosed during four health examinations (0 to 20 times)

The number of meeting individual components	No. of participants	AF	IR (1000PY)	HR (95% CI)		
				Model1	Model2	Model3
0	278,704	549	0.37	1.00 (Reference)	1.00 (Reference)	1.00 (Reference)
1	297,650	729	0.37	1.25 (1.12–1.39)	1.03 (0.92–1.15)	1.04 (0.93–1.16)
2	283,079	939	0.63	1.69 (1.52–1.88)	1.21 (1.09–1.34)	1.24 (1.11–1.38)
3	265,009	1007	0.72	1.94 (1.75–2.15)	1.23 (1.11–1.36)	1.27 (1.14–1.41)
4	263,875	1345	0.97	2.61 (2.36–2.88)	1.43 (1.30–1.58)	1.49 (1.35–1.65)
5	235,071	1342	1.08	2.92 (2.65–3.23)	1.47 (1.33–1.62)	1.54 (1.39–1.71)
6	210,455	1397	1.26	3.41 (3.09–3.76)	1.59 (1.44–1.75)	1.68 (1.52–1.87)
7	187,332	1427	1.45	3.92 (3.55–4.32)	1.72 (1.56–1.90)	1.84 (1.66–2.04)
8	173,024	1407	1.55	4.18 (3.79–4.62)	1.74 (1.58–1.93)	1.87 (1.68–2.08)
9	144,294	1282	1.70	4.58 (4.15–5.06)	1.85 (1.67–2.05)	2.00 (1.79–2.23)
10	122,156	1179	1.85	4.99 (4.50–5.52)	1.94 (1.75–2.15)	2.10 (1.88–2.34)
11	100,785	1046	1.99	5.37 (4.84–5.96)	2.03 (1.82–2.25)	2.20 (1.96–2.46)
12	89,005	980	2.11	5.70 (5.13–6.33)	2.07 (1.86–2.30)	2.24 (2.00–2.51)
13	68,103	855	2.41	6.51 (5.85–7.25)	2.29 (2.06–2.56)	2.49 (2.21–2.80)
14	52,236	723	2.66	7.19 (6.43–8.03)	2.46 (2.19–2.75)	2.66 (2.35–3.00)
15	38,764	572	2.84	7.67 (6.82–8.62)	2.57 (2.29–2.90)	2.78 (2.44–3.16)
16	32,590	556	3.29	8.88 (7.89–9.99)	2.81 (2.50–3.17)	3.04 (2.66–3.46)
17	17,981	316	3.39	9.16 (7.98–10.52)	2.87 (2.50–3.30)	3.03 (2.61–3.52)
18	11,711	204	3.36	9.07 (7.73–10.66)	2.81 (2.39–3.30)	2.93 (2.46–3.48)
19	7451	136	3.52	9.50 (7.87–11.46)	2.94 (2.43–3.55)	3.03 (2.48–3.69)
20	5914	121	3.97	10.73 (8.81–13.06)	3.15 (2.58–3.84)	3.11 (2.52–3.83)
				p < 0.001	p < 0.001	p < 0.001

Model 1 is unadjusted

Model 2 is adjusted for age, sex, smoking status, alcohol intake, regular exercise, and low income

Model 3 is adjusted for age, sex, smoking status, alcohol intake, regular exercise, low income, waist circumference, systolic blood pressure, fasting glucose, logarithm of TG, and HDL-C level

*AF *atrial fibrillation, *IR* incidence rate, *PY* person-years, *HR* hazard ratio, *CI* confidence interval

### Temporal trends of metabolic burden and the risk of AF

**Fig. 4:**
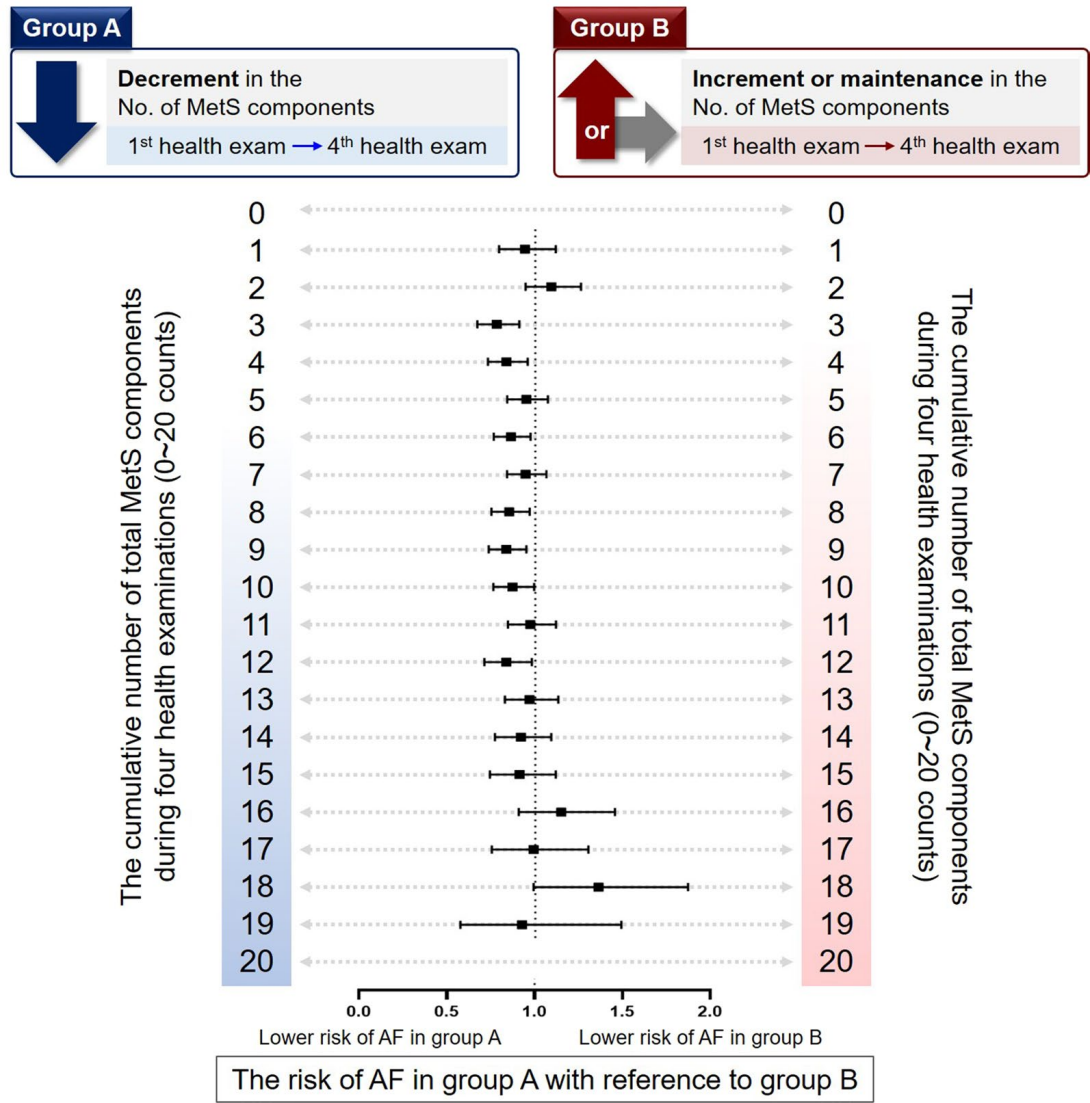
The risk of atrial fibrillation according to the temporal trends in metabolic burden. *No* number, *MetS* metabolic syndrome

We compared the risk of AF according to the temporal trends in metabolic burden (Fig. 4 and Additional file 1: Table S2). From 3 to 12 counts of the total cumulative metabolic components during the four health examinations, group A (with a decrease in the number of MetS components) exhibited a lower risk of AF than group B (with an increase or maintenance in the number of MetS components). Those with less than 3 or more than 12 MetS components presented insignificant differences in the risk of AF regarding the temporal change in metabolic burden.

### Subgroup and sensitivity analysis

Results of subgroup analyses of the association between AF risk and the cumulative number of MetS components, according to sex and obesity, are shown in Additional file 1: Table S3 and Table S4, respectively. The incidence of AF did not show an interaction with sex or the presence of obesity (*P*-for-interaction 0.25 and 0.77, respectively).

In sensitivity analysis excluding participants with AF diagnosed in the first 2 years of follow-up, the progressive increase in the risk of AF according to the cumulative number of all MetS components was consistent with the main results (Additional file 1: Table S5).

## Discussion

To the best of our knowledge, this is the first study to report the cumulative burden of MetS and its components on the risk of AF in a large nationwide population-based cohort study. We demonstrated several principal findings, which are summarized as follows: (1) the cumulative burden of MetS during the four health examinations had a linear correlation with the risk of AF; (2) the cumulative burden of each MetS component showed a positive association with the risk of AF; (3) among the five metabolic components, elevated blood pressure had the greatest increase in AF risk; (4) the cumulative number of total MetS components showed an incremental association with the risk of AF; and (5) temporal trends in MetS burden had a different effect on the risk of AF.

Both MetS and AF confer a high burden of cardiovascular morbidity and mortality, and the association between MetS and AF has been reported in various studies [10, 11, 26, 27]. Compelling evidence supports that MetS itself is associated with the development of AF, and the presence of an increasing number of MetS components predisposes individuals to a higher risk of AF [11, 26, 28–30]. However, these studies analyzed the number of MetS components at a single point. In contrast to previous studies, we have investigated the influence of temporal accumulation of MetS by combining four serial health examinations collectively. While participants diagnosed with MetS once had an 18% increased risk of AF compared to that in non-MetS participants, those diagnosed with MetS repeatedly at four times of measurement had a 72% higher risk of AF. Notably, the risk of AF increased by 3.1-fold as the number of fulfilled MetS components increased to a total of 20 counts during consecutive health examinations. Such a positive increase in AF according to a repetitive diagnosis of metabolic components might suggest a dose–response (or temporal accumulation) perspective in evaluating the hazards of cardiovascular diseases. This finding is noticeable because it implicates that the risk of AF development is different among patients with metabolic derangements depending on the past and upcoming metabolic burden. Given the proportional increase in AF risk according to the degree of metabolic burden integrating temporal changes, it is plausible to infer that the risk of AF would be higher in those diagnosed with MetS more than four times during their health examination checkups.

Regarding the relationship between elevated TG and the risk of AF, there were controversial reports [11, 26, 28, 31, 32]. The Niigata Preventive Medicine Study and post-hoc analysis from the ARIC study reported that hypertriglyceridemia was not related to incident AF [11, 26]. In addition, a recent study reported no association between elevated TG levels and the risk of AF [32]. However, the Multi-Ethnic Study of Atherosclerosis (MESA) and the Framingham Heart Study (FHS) reported that hypertriglyceridemia was associated with a higher risk of AF [31]. In our study, meeting the criteria of elevated TG once or twice during 4 times of health screening examinations seems insignificantly or minimally associated with the risk of AF. Nonetheless, the association became evident as elevated TG levels were repeatedly checked, whereby the risk of AF was as high as 27% if hypertriglyceridemia was repeatedly confirmed through the entire health examinations of the study period, even after adjusting for potential confounders and other MetS components. Elevated TG is reported to be associated with endothelial dysfunction and the presence of microvascular disease [33, 34], which could be an early manifestation of atherosclerosis. Moreover, it is related to an increased risk of recurrent coronary artery disease [35], as well as insulin resistance, and is known to increase blood glucose levels [36]. Although the exact mechanism of the development of AF is not known, the pro-atherogenic property of elevated TG might play a role in AF development.

Along with hypertriglyceridemia, other metabolic components and MetS itself could be linked to a higher risk of AF by several pathophysiological mechanisms. Mechanical alterations including an increase in atrial size and ventricular hypertrophy due to MetS, obesity, hypertension, and low HDL-C may contribute to the development of AF [14, 37–40]. Atrial interstitial fibrosis, abnormal calcium homeostasis, and inappropriate neurohumoral activation led by insulin resistance are also reported to increased AF susceptibility [41]. Moreover, elevated levels of inflammation and oxidative stress have been proposed in the pathogenesis of both MetS and AF; indeed, HDL-C exerts anti-inflammatory and anti-oxidative activities that promote vascular health; hence, low HDL-C levels are related to a proinflammatory milieu [42–44]. These metabolic changes may predispose patients with MetS or individual metabolic components to AF development. Meanwhile, elevated BP and low HDL-C levels have the most prominent increase in AF risk, which is consistent with prior research [11, 27]. Impaired fasting glucose showed only a modest increase in AF development, consistent with a recent study evaluating the impact of ‘exposure’ to metabolic syndrome on the increased risk of MI and stroke, both sharing common predisposing factors with AF [21]. Although the exact underlying mechanisms explaining the differences in magnitudes of AF risk among metabolic components are not answered in the present study, we could hypothesize that elevated BP and adverse lipid profiles play more pivotal roles on substrate formation for AF development. Also, as the prominent increase in the risk of AF according to repeated checks of impaired fasting glucose was attenuated after adjusting for other covariates, it might still contribute to the development of AF as a cluster of metabolic diseases rather than carry a sole dominant effect.

The International Diabetes Federation (IDF) estimates that approximately 25% of the world's population, which corresponds to over a billion people, is affected by MetS despite the fact that the prevalence varies widely according to age, ethnicity, and sex of the population studied [45, 46]. Considering that AF is strongly associated with cardiovascular diseases such as stroke, heart failure, myocardial infarction, dementia, and increased mortality [3, 47], exploring the pragmatic association between metabolic derangements and AF along with the understanding of potential subsequent diseases is important to address the public health care burden of AF and AF-related complications. Our large study population with a mean age at mid-forties, much younger than that reported in previous studies [11, 26, 27], thereby representing the most active group socioeconomically, contributes to emphasize the awareness of the group harboring the risk of AF and the early initiation of prevention strategies. Importantly, we ascertained that even meeting one or two components of MetS over time is sufficient to increase the risk of AF, and thus, it would be crucial to make a concerted effort to minimize the metabolic derangements.

Even within the participants who presented the same cumulative number of total MetS components in the four health examinations, each may carry a different risk of AF depending on the change in metabolic burden. Those with a decreased number of MetS components over time showed a lower risk of AF than participants with an increase or maintenance in the number of MetS components at the last health examination compared to the first examination. Given that recovery from MetS is known to be significantly associated with a decreased risk of major adverse cardiovascular events [48], our results are consistent with previous findings by focusing on the most common cardiac rhythm disorder, AF. Therefore, it would be important to manage MetS component(s) to mitigate the risk of AF, and this management should be considered as part of the overall management strategy for preventing AF [49, 50]. Indeed, lifestyle modification (including addressing many risk factors within the MetS components) is part of the atrial fibrillation better care (ABC) pathway for holistic AF care, which is advocated in the new 2020 ESC guidelines [51].

### Limitations

Our study has several limitations. First, a considerable proportion of the study population already had diabetes mellitus and hypertension, which are evident and strong risk factors for AF. Instead, we show the risk gradient of AF by investigating the cumulative burden of metabolic derangements in a large general population at a relatively young age. Second, more than two-thirds of the study participants were men because employees are more likely to undergo regular health examinations provided at work places; hence, selection bias of sex might be introduced. However, subgroup analysis did not show any significant interaction with sex. Third, the degree and the relationship of AF risk with metabolic burden may differ in western populations because our study was conducted in an Asian population. Fourth, we incorporated many covariates including lifestyle behaviors, but unavailable confounding and unmeasured factors such as the presence of other inflammatory diseases or the measure of left atrial size could not be fully adjusted. Fifth, the metabolic status may change during the follow-up period and mitigate the risk. Lastly, the causality and underlying mechanistic link between MetS and AF were not answered. Nevertheless, we expanded the previous understanding of the association between MetS and AF by showing the biological gradient of metabolic derangements and AF risk in this large population-based study with serial health examination data.

## Conclusions

In this large Asian population-based cohort, the cumulative burden of MetS diagnostic criteria and its components over time has a positive correlation with the risk of incident AF. Given the close association between the cumulative number of total MetS components and the risk of AF, maximal effort to detect and correct metabolic derangements even before the development of MetS might be important to prevent AF and related cardiovascular diseases.

## Supplementary Information

**Additional file 1.** Additional figure and tables.

## Data Availability

The datasets used and analysed during the current study are available from the corresponding author on reasonable request.

## Abbreviations

AF: Atrial fibrillation
MI: Myocardial infarction
MetS: Metabolic syndrome
HDL-C: High-density lipoprotein cholesterol
TG: Triglyceride
NHID: National Health Information Database
WC: Waist circumference
HR: Hazard ratio
CI: Confidence interval
