# Supplementary material for: Cumulative burden of metabolic syndrome and its components on the risk of atrial fibrillation: a nationwide population-based study
Source: Cardiovasc Diabetol. 2021 Jan 19;20:20. doi: 10.1186/s12933-021-01215-8 (PMC7816376; doi:10.1186/s12933-021-01215-8)
Supplement: Supplementary file 1 — Additional file 1. Additional figure and tables. [file 12933_2021_1215_MOESM1_ESM.docx]

**Additional Fig. and Table**

**Fig. S1.** Selection of study population from the national health insurance data service.

**Table S1**. Definitions of covariates and outcomes.

**Table S2**. The risk of atrial fibrillation according to the temporal trends in metabolic burden and the cumulative number of total MetS components diagnosed during four health examinations.

**Table S3**. The risk of atrial fibrillation according to the sex and the cumulative number of total MetS components diagnosed during four health examinations.

**Table S4**. The risk of atrial fibrillation according to the presence of obesity and the cumulative number of total MetS components diagnosed during four health examinations.

**Table S5.** The risk of atrial fibrillation after the first 2 years of follow-up according to the cumulative number of total MetS components diagnosed during four health examinations.

**Fig. S1.** Selection of study population from the national health insurance data service.


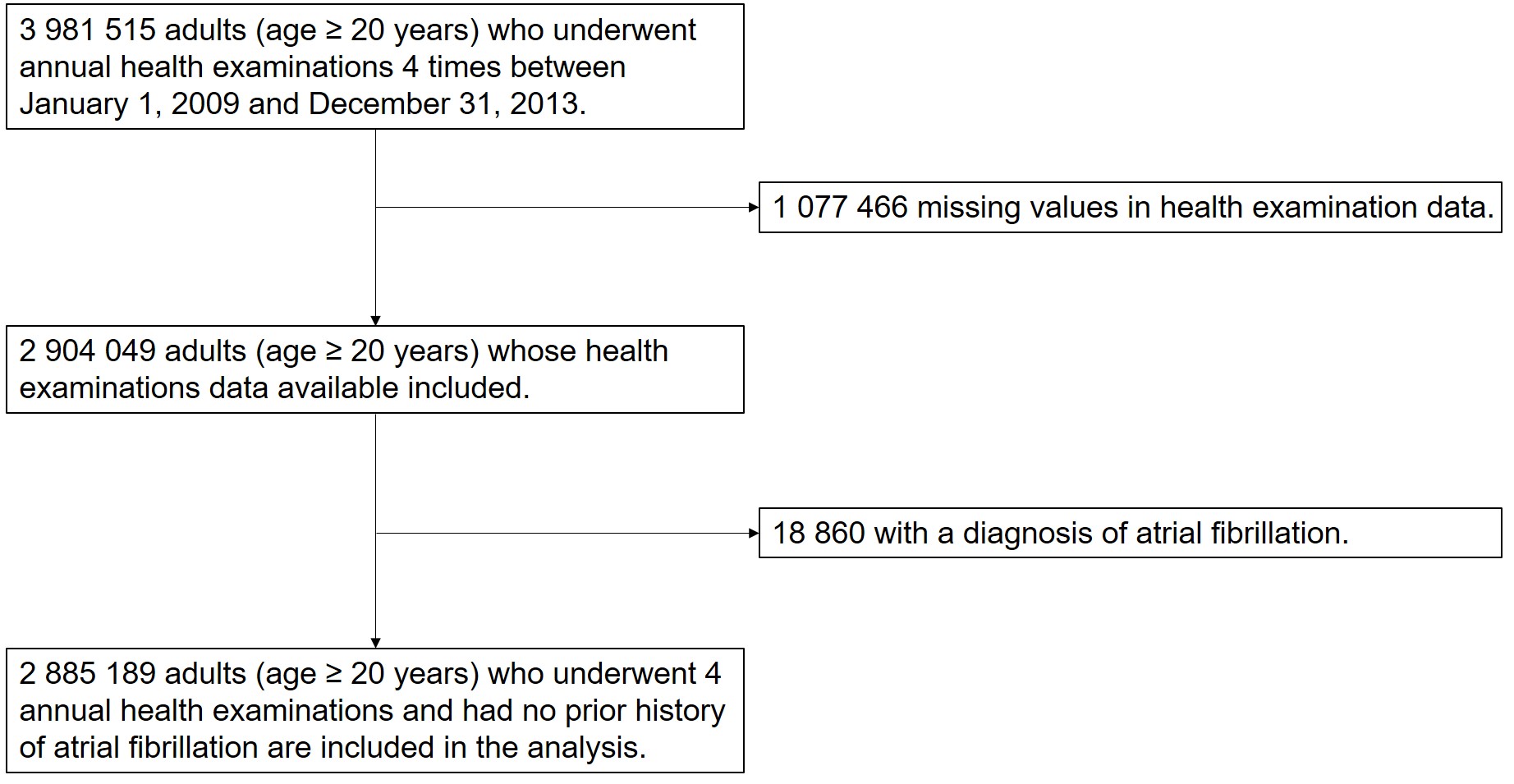


**Table S1**. Definitions of covariates and outcomes.

| **Diagnosis** | **ICD-10-CM code and medication** | **Number of diagnosis** | **Diagnosis test or treatment** | **Combination*** |
| --- | --- | --- | --- | --- |
| **Inclusion/Exclusion** |  |  |  |  |
| Atrial fibrillation | I48.0-48.4, I48.9 | Admission ≥ 1 or outpatient department ≥ 2 | N/A | 1+2 |
| Valvular atrial fibrillation | I05.0, I05.2, I05.9, Z95.2-Z95.4 | Admission ≥ 1 or outpatient department ≥ 1 | N/A | 1+2 |
| **Comorbidities based on the last health examination** | | |  |  |
| **Comorbidities** |  |  |  |  |
| Hypertension | I10-I13, I15; and minimum 1 prescription of anti-hypertensive drug (thiazide, loop diuretics, aldosterone antagonist, alpha-/beta-blocker, calcium-channel blocker, angiotensin-converting enzyme inhibitor, or angiotensin II receptor blocker) | Admission ≥ 1 or outpatient department ≥ 2 | Systolic/diastolic blood pressure ≥ 140/90 mmHg | 1+2 or 3 |
| Diabetes mellitus | E11-E14; and minimum 1 prescription of anti-diabetic drugs (sulfonylureas, metformin, meglitinides, thiazolidinediones, dipeptidyl peptidase-4 inhibitors, α-glucosidase inhibitors, or insulin) | Admission ≥ 1 or outpatient department ≥ 2 | Fasting glucose level ≥ 126 mg/dL | 1+2 or 3 |
| Dyslipidemia | E78 | Admission ≥ 1 or outpatient department ≥ 1 | Total cholesterol ≥ 240 mg/dL | 1+2 or 3 |
| MI | I21, I22 | Admission ≥ 1 or outpatient department ≥ 1 | N/A | 1+2 |
| HF | I50 | Admission ≥ 1 or outpatient department ≥ 1 | N/A | 1+2 |
| PAD | I70, I73 | Admission ≥ 1 or outpatient department ≥ 1 | N/A | 1+2 |
| COPD | J41-44 | Admission ≥ 1 | N/A | 1+2 |
| CKD | N/A | N/A | eGFR<60ml/min/1.73m^2^ | 3 |
| Cancer | C00-97 and RID code (V193) | Admission ≥ 1 or outpatient department ≥ 1 | N/A | 1+2 |
| **Definitions of life style behavior based on the last health examination questionnaire** | | | | |
| **Alcohol consumption** |  |  |  |  |
| Mild to moderate drinker | Alcohol consumption > 0g to < 30g per day | |  |  |
| Heavy drinker | Alcohol consumption ≥ 30g per day | |  |  |
| **Smoking** |  |  |  |  |
| Ex-smoker | Ex-smoker at the 1st examination and sustaining non-smoking till the 2nd examination | |  |  |
| Current smoker | Current smoker at the 2nd examination regardless of the smoking status at the 1st examination. | | | |
| **Information of income** | | | | |
| Low income | Income belongs to lower 20% among the entire Korean population and supported by the Medical Aid program | | |  |

Abbreviation: N/A, not applicable; MI, myocardial infarction; HF, heart failure; PAD, peripheral artery disease; COPD, chronic obstructive pulmonary disease; CKD, chronic kidney disease.

* Combination: 1= ICD-10-CM code and medication; 2 = Number of diagnosis; and 3 = Diagnosis test or treatment

**Table S2**. The risk of atrial fibrillation according to the temporal change of number fulfilling MetS components and the cumulative number of total MetS components diagnosed during four health examinations.

| The number of meeting individual components | **Decreased number of**  **metabolic syndrome components (Group A)** | | | **Increased or maintained number of**  **metabolic syndrome components (Group B)** | | | **The HR (95% CI) of AF**  **in Group A with reference to Group B** | |
| --- | --- | --- | --- | --- | --- | --- | --- | --- |
|  | No. of participants | Events (AF) | IR (1000PY) | No. of participants | Events (AF) | IR (1000PY) |  |  |
| 0 | 278725 | 541 | 0.36 | 278725 | 541 | 0.37 | N/A | p-value |
| 1 | 79164 | 181 | 0.43 | 218508 | 541 | 0.47 | 0.94 (0.79 - 1.12) | 0.49 |
| 2 | 88140 | 309 | 0.66 | 194959 | 614 | 0.60 | 1.09 (0.95 - 1.26) | 0.23 |
| 3 | 79918 | 255 | 0.60 | 185110 | 733 | 0.75 | 0.78 (0.67 - 0.91) | 0.00 |
| 4 | 66681 | 290 | 0.82 | 197221 | 1039 | 1.00 | 0.84 (0.73 - 0.96) | 0.01 |
| 5 | 70381 | 395 | 1.06 | 164711 | 930 | 1.07 | 0.95 (0.84 - 1.07) | 0.40 |
| 6 | 64396 | 394 | 1.16 | 146070 | 984 | 1.28 | 0.86 (0.76 - 0.97) | 0.02 |
| 7 | 54953 | 419 | 1.45 | 132401 | 999 | 1.44 | 0.95 (0.84 - 1.06) | 0.35 |
| 8 | 44050 | 319 | 1.38 | 128996 | 1066 | 1.58 | 0.85 (0.75 - 0.97) | 0.01 |
| 9 | 41433 | 330 | 1.52 | 102878 | 937 | 1.74 | 0.84 (0.74 - 0.95) | 0.01 |
| 10 | 34954 | 308 | 1.68 | 87213 | 853 | 1.87 | 0.87 (0.76 - 0.99) | 0.04 |
| 11 | 26989 | 283 | 2.01 | 73804 | 746 | 1.94 | 0.97 (0.85 - 1.12) | 0.69 |
| 12 | 19357 | 185 | 1.83 | 69664 | 785 | 2.16 | 0.84 (0.71 - 0.98) | 0.03 |
| 13 | 17510 | 213 | 2.34 | 50598 | 627 | 2.38 | 0.97 (0.83 - 1.13) | 0.68 |
| 14 | 13273 | 170 | 2.46 | 38975 | 543 | 2.68 | 0.92 (0.77 - 1.09) | 0.33 |
| 15 | 8806 | 118 | 2.57 | 29969 | 448 | 2.88 | 0.91 (0.74 - 1.12) | 0.37 |
| 16 | 4494 | 82 | 3.51 | 28099 | 464 | 3.18 | 1.15 (0.91 - 1.45) | 0.25 |
| 17 | 3985 | 67 | 3.24 | 14002 | 245 | 3.38 | 0.99 (0.75 - 1.30) | 0.94 |
| 18 | 2711 | 57 | 4.06 | 9002 | 143 | 3.06 | 1.36 (0.99 - 1.87) | 0.06 |
| 19 | 1433 | 23 | 3.10 | 6020 | 111 | 3.55 | 0.93 (0.57 - 1.49) | 0.75 |
| 20 | 5915 | 118 | 3.87 | 5915 | 118 | 3.87 | N/A |  |

Abbreviation: AF, atrial fibrillation; IR, incidence rate; PY, person-years; HR, hazard ratio; CI, confidence interval.

**Table S3**. The risk of atrial fibrillation according to the sex and the cumulative number of total MetS components diagnosed during four health examinations.

| The number of meeting individual components | **Male** | | | | | | **Female** | | | | | |
| --- | --- | --- | --- | --- | --- | --- | --- | --- | --- | --- | --- | --- |
|  | No. of participants | AF | IR  (1000PY) | HR (95% CI) | | | No. of participants | AF | IR  (1000PY) | HR (95% CI) | | |
|  |  |  |  | Model 1 | Model 2 | Model 3 |  |  |  | Model 1 | Model 2 | Model 3 |
| 0 | 118335 | 338 | 0.54 | 1.00 (Reference) | 1.00 (Reference) | 1.00 (Reference) | 160369 | 211 | 0.25 | 1.00 (Reference) | 1.00 (Reference) | 1.00 (Reference) |
| 1 | 164992 | 530 | 0.60 | 1.13 (0.98 - 1.29) | 1.08 (0.94 - 1.24) | 1.10 (0.96 - 1.26) | 132658 | 199 | 0.28 | 1.15 (0.94 - 1.39) | 0.92 (0.76 - 1.12) | 0.92 (0.76 - 1.12) |
| 2 | 180562 | 739 | 0.77 | 1.44 (1.27 - 1.64) | 1.28 (1.13 - 1.46) | 1.32 (1.16 - 1.51) | 102517 | 200 | 0.37 | 1.49 (1.23 - 1.81) | 1.02 (0.84 - 1.24) | 1.01 (0.83 - 1.23) |
| 3 | 184921 | 793 | 0.81 | 1.51 (1.33 - 1.72) | 1.25 (1.10 - 1.42) | 1.31 (1.15 - 1.49) | 80088 | 214 | 0.51 | 2.05 (1.69 - 2.48) | 1.20 (0.99 - 1.45) | 1.18 (0.97 - 1.44) |
| 4 | 193402 | 1090 | 1.07 | 1.99 (1.76 - 2.25) | 1.46 (1.29 - 1.65) | 1.54 (1.36 - 1.75) | 70473 | 255 | 0.69 | 2.78 (2.32 - 3.34) | 1.39 (1.15 - 1.67) | 1.37 (1.13 - 1.66) |
| 5 | 181282 | 1120 | 1.17 | 2.19 (1.94 - 2.47) | 1.50 (1.33 - 1.69) | 1.60 (1.42 - 1.82) | 53789 | 222 | 0.79 | 3.18 (2.63 - 3.83) | 1.40 (1.16 - 1.70) | 1.38 (1.13 - 1.69) |
| 6 | 167043 | 1162 | 1.32 | 2.47 (2.19 - 2.79) | 1.60 (1.41 - 1.80) | 1.73 (1.52 - 1.95) | 43412 | 235 | 1.04 | 4.18 (3.47 - 5.03) | 1.66 (1.37 - 2.01) | 1.62 (1.32 - 1.99) |
| 7 | 152765 | 1225 | 1.53 | 2.85 (2.53 - 3.21) | 1.77 (1.57 - 1.99) | 1.93 (1.70 - 2.19) | 34567 | 202 | 1.12 | 4.52 (3.73 - 5.49) | 1.64 (1.34 - 2.00) | 1.60 (1.29 - 1.98) |
| 8 | 141532 | 1233 | 1.66 | 3.10 (2.75 - 3.50) | 1.82 (1.61 - 2.05) | 2.00 (1.76 - 2.27) | 31492 | 174 | 1.06 | 4.27 (3.50 - 5.22) | 1.45 (1.18 - 1.79) | 1.42 (1.14 - 1.78) |
| 9 | 119311 | 1110 | 1.78 | 3.32 (2.94 - 3.75) | 1.91 (1.69 - 2.15) | 2.11 (1.85 - 2.40) | 24983 | 172 | 1.33 | 5.35 (4.37 - 6.54) | 1.69 (1.37 - 2.08) | 1.65 (1.32 - 2.07) |
| 10 | 101675 | 1011 | 1.90 | 3.55 (3.14 - 4.01) | 1.98 (1.75 - 2.24) | 2.19 (1.92 - 2.50) | 20481 | 168 | 1.58 | 6.39 (5.21 - 7.82) | 1.88 (1.53 - 2.33) | 1.83 (1.45 - 2.31) |
| 11 | 84765 | 926 | 2.09 | 3.91 (3.45 - 4.43) | 2.12 (1.87 - 2.40) | 2.36 (2.06 - 2.69) | 16020 | 120 | 1.45 | 5.85 (4.68 - 7.32) | 1.67 (1.33 - 2.11) | 1.62 (1.26 - 2.10) |
| 12 | 73404 | 864 | 2.25 | 4.21 (3.71 - 4.77) | 2.18 (1.92 - 2.48) | 2.43 (2.12 - 2.78) | 15601 | 116 | 1.44 | 5.81 (4.63 - 7.28) | 1.58 (1.25 - 2.00) | 1.54 (1.19 - 1.99) |
| 13 | 56494 | 733 | 2.49 | 4.65 (4.08 - 5.28) | 2.35 (2.06 - 2.68) | 2.62 (2.28 - 3.01) | 11609 | 122 | 2.04 | 8.25 (6.60 - 10.31) | 2.16 (1.71 - 2.72) | 2.08 (1.61 - 2.69) |
| 14 | 43454 | 632 | 2.79 | 5.22 (4.57 - 5.95) | 2.57 (2.25 - 2.93) | 2.86 (2.47 - 3.30) | 8782 | 91 | 2.01 | 8.13 (6.34 - 10.39) | 2.02 (1.57 - 2.61) | 1.94 (1.47 - 2.57) |
| 15 | 32396 | 491 | 2.91 | 5.44 (4.73 - 6.24) | 2.64 (2.30 - 3.04) | 2.94 (2.53 - 3.41) | 6368 | 81 | 2.48 | 10.00 (7.74 - 12.92) | 2.38 (1.83 - 3.10) | 2.27 (1.69 - 3.05) |
| 16 | 26877 | 471 | 3.37 | 6.29 (5.47 - 7.24) | 2.86 (2.48 - 3.29) | 3.18 (2.73 - 3.70) | 5713 | 85 | 2.91 | 11.76 (9.14 - 15.13) | 2.76 (2.12 - 3.58) | 2.62 (1.96 - 3.50) |
| 17 | 14789 | 277 | 3.6 | 6.73 (5.75 - 7.89) | 3.04 (2.59 - 3.56) | 3.30 (2.78 - 3.92) | 3192 | 39 | 2.39 | 9.68 (6.88 - 13.63) | 2.17 (1.53 - 3.08) | 2.03 (1.39 - 2.95) |
| 18 | 9686 | 182 | 3.61 | 6.75 (5.64 - 8.08) | 3.02 (2.52 - 3.62) | 3.23 (2.67 - 3.91) | 2025 | 22 | 2.13 | 8.61 (5.55 - 13.35) | 1.90 (1.22 - 2.97) | 1.74 (1.09 - 2.79) |
| 19 | 6169 | 120 | 3.73 | 6.98 (5.67 - 8.60) | 3.12 (2.53 - 3.84) | 3.31 (2.66 - 4.12) | 1282 | 16 | 2.44 | 9.89 (5.95 - 16.43) | 2.16 (1.29 - 3.61) | 1.96 (1.14 - 3.35) |
| 20 | 4791 | 101 | 4.07 | 7.62 (6.10 - 9.51) | 3.18 (2.54 - 3.97) | 3.23 (2.56 - 4.08) | 1123 | 20 | 3.51 | 14.24 (9.01 - 22.52) | 3.15 (1.98 - 5.01) | 2.74 (1.68 - 4.49) |
|  |  |  |  | p < 0.001 | p < 0.001 | p < 0.001 |  |  |  | p < 0.001 | p < 0.001 | p < 0.001 |

Abbreviation: AF, atrial fibrillation; IR, incidence rate; PY, person-years; HR, hazard ratio; CI, confidence interval.

Model 1 is unadjusted.

Model 2 is adjusted for age, sex, smoking status, alcohol intake, regular exercise, and low income.

Model 3 is adjusted for age, sex, smoking status, alcohol intake, regular exercise, low income, waist circumference, systolic blood pressure, fasting glucose, logarithm of TG, and HDL-C level.

Model 1 *P* for interaction < 0.001,

Model 2 *P* for interaction = 0.252

Model 3 *P* for interaction = 0.2503

**Table S4**. The risk of atrial fibrillation according to the presence of obesity and the cumulative number of total MetS components diagnosed during four health examinations.

| The number of meeting individual components | **Subjects without Obesity** | | | | | | | **Subjects with Obesity** | | | | | | |
| --- | --- | --- | --- | --- | --- | --- | --- | --- | --- | --- | --- | --- | --- | --- |
|  | BMI | No. of participants | AF | IR  (1000PY) | HR (95% CI) | | | BMI | No. of participants | AF | IR  (1000PY) | HR (95% CI) | | |
|  |  |  |  |  | Model 1 | Model 2 | Model 3 |  |  |  |  | Model 1 | Model 2 | Model 3 |
| 0 | 20.93 ± 2.00 | 263494 | 519 | 0.37 | 1.00 (Reference) | 1.00 (Reference) | 1.00 (Reference) | 26.03 ± 0.97 | 15210 | 30 | 0.37 | 1.00 (Reference) | 1.00 (Reference) | 1.00 (Reference) |
| 1 | 21.44 ± 1.97 | 267262 | 646 | 0.46 | 1.23 (1.10 - 1.38) | 1.01 (0.90 - 1.13) | 1.02 (0.91 - 1.15) | 26.18 ± 1.09 | 30388 | 83 | 0.52 | 1.39 (0.91 - 2.11) | 1.29 (0.85 - 1.97) | 1.31 (0.86 - 1.98) |
| 2 | 21.80 ± 1.92 | 240197 | 805 | 0.63 | 1.71 (1.53 - 1.91) | 1.19 (1.07 - 1.33) | 1.23 (1.10 - 1.38) | 26.32 ± 1.21 | 42882 | 134 | 0.59 | 1.59 (1.07 - 2.36) | 1.38 (0.93 - 2.05) | 1.40 (0.94 - 2.08) |
| 3 | 22.08 ±1.85 | 211769 | 839 | 0.75 | 2.02 (1.81 - 2.26) | 1.23 (1.10 - 1.37) | 1.28 (1.15 - 1.44) | 26.44 ± 1.30 | 53240 | 168 | 0.60 | 1.61 (1.09 - 2.37) | 1.30 (0.88 - 1.92) | 1.34 (0.90 - 1.97) |
| 4 | 22.20 ± 1.80 | 198472 | 1061 | 1.01 | 2.73 (2.46 - 3.04) | 1.41 (1.27 - 1.57) | 1.49 (1.34 - 1.66) | 26.58 ± 1.45 | 65403 | 284 | 0.82 | 2.21 (1.52 - 3.23) | 1.60 (1.10 - 2.34) | 1.66 (1.14 - 2.42) |
| 5 | 22.49 ± 1.73 | 163131 | 983 | 1.15 | 3.09 (2.77 - 3.43) | 1.42 (1.28 - 1.59) | 1.53 (1.36 - 1.71) | 26.74 ± 1.58 | 71940 | 359 | 0.95 | 2.55 (1.76 - 3.70) | 1.71 (1.18 - 2.48) | 1.78 (1.22 - 2.59) |
| 6 | 22.66 ± 1.67 | 133976 | 966 | 1.37 | 3.70 (3.33 - 4.12) | 1.55 (1.39 - 1.73) | 1.69 (1.51 - 1.89) | 26.90 ± 1.72 | 76479 | 431 | 1.07 | 2.88 (1.99 - 4.17) | 1.80 (1.24 - 2.60) | 1.88 (1.29 - 2.73) |
| 7 | 22.79 ± 1.62 | 108320 | 928 | 1.63 | 4.41 (3.96 - 4.91) | 1.71 (1.53 - 1.90) | 1.88 (1.68 - 2.12) | 27.08 ± 1.84 | 79012 | 499 | 1.20 | 3.24 (2.24 - 4.68) | 1.90 (1.31 - 2.75) | 2.00 (1.38 - 2.89) |
| 8 | 22.85 ± 1.59 | 92665 | 850 | 1.75 | 4.72 (4.24 - 5.27) | 1.70 (1.52 - 1.91) | 1.901 (1.69 - 2.14) | 27.26 ± 1.97 | 80359 | 557 | 1.32 | 3.56 (2.46 - 5.14) | 1.95 (1.35 - 2.82) | 2.06 (1.42 - 2.98) |
| 9 | 23.01 ± 1.52 | 68127 | 646 | 1.81 | 4.90 (4.36 - 5.50) | 1.68 (1.49 - 1.89) | 1.91 (1.68 - 2.17) | 27.44 ± 2.07 | 76167 | 636 | 1.60 | 4.29 (2.98 - 6.19) | 2.24 (1.55 - 3.23) | 2.37 (1.64 - 3.43) |
| 10 | 23.11 ± 1.47 | 51529 | 576 | 2.14 | 5.78 (5.13 - 6.51) | 1.88 (1.66 - 2.12) | 2.15 (1.89 - 2.45) | 27.62 ± 2.18 | 70627 | 603 | 1.63 | 4.40 (3.05 - 6.34) | 2.18 (1.51 - 3.14) | 2.31 (1.59 - 3.35) |
| 11 | 23.20 ± 1.42 | 37282 | 451 | 2.32 | 6.27 (5.52 - 7.11) | 1.94 (1.70 - 2.20) | 2.25 (1.95 - 2.58) | 27.80 ± 2.25 | 63503 | 595 | 1.80 | 4.84 (3.35 - 6.98) | 2.29 (1.59 - 3.31) | 2.43 (1.68 - 3.53) |
| 12 | 23.14 ± 1.46 | 31589 | 416 | 2.53 | 6.84 (6.01 - 7.78) | 2.02 (1.77 - 2.30) | 2.33 (2.03 - 2.69) | 27.97 ± 2.36 | 57416 | 564 | 1.88 | 5.07 (3.51 - 7.31) | 2.29 (1.59 - 3.31) | 2.44 (1.68 - 3.55) |
| 13 | 23.25 ± 1.39 | 20878 | 319 | 2.94 | 7.94 (6.91 - 9.13) | 2.22 (1.93 - 2.56) | 2.56 (2.20 - 2.98) | 28.11 ± 2.40 | 47225 | 536 | 2.18 | 5.87 (4.06 - 8.47) | 2.56 (1.77 - 3.70) | 2.74 (1.88 - 3.99) |
| 14 | 23.30 ± 1.38 | 14147 | 233 | 3.17 | 8.56 (7.34 - 9.99) | 2.28 (1.95 - 2.67) | 2.621 (2.22 - 3.10) | 28.24 ± 2.44 | 38089 | 490 | 2.47 | 6.66 (4.61 - 9.63) | 2.79 (1.93 - 4.04) | 2.99 (2.05 - 4.36) |
| 15 | 23.39 ± 1.35 | 9011 | 151 | 3.23 | 8.73 (7.29 - 10.47) | 2.30 (1.91 - 2.76) | 2.65 (2.18 - 3.21) | 28.35 ± 2.46 | 29753 | 421 | 2.72 | 7.33 (5.06 - 10.61) | 2.95 (2.03 - 4.27) | 3.16 (2.17 - 4.62) |
| 16 | 23.19 ± 1.43 | 8730 | 179 | 3.97 | 10.73 (9.05 - 12.71) | 2.62 (2.20 - 3.11) | 2.99 (2.49 - 3.59) | 28.45 ± 2.53 | 23860 | 377 | 3.04 | 8.19 (5.65 - 11.88) | 3.19 (2.20 - 4.63) | 3.41 (2.33 - 4.99) |
| 17 | 23.87 ± 0.99 | 2457 | 50 | 3.96 | 10.71 (8.01 - 14.32) | 2.35 (1.75 - 3.14) | 2.60 (1.93 - 3.51) | 28.58 ± 2.54 | 15524 | 266 | 3.30 | 8.90 (6.10 - 12.98) | 3.29 (2.25 - 4.81) | 3.49 (2.37 - 5.14) |
| 18 | 24.00 ± 0.91 | 1130 | 32 | 5.57 | 15.10 (10.57 - 21.58) | 3.23 (2.26 - 4.62) | 3.55 (2.46 - 5.11) | 28.72 ± 2.53 | 10581 | 172 | 3.13 | 8.43 (5.72 - 12.42) | 3.02 (2.05 - 4.45) | 3.15 (2.12 - 4.69) |
| 19 | 24.10 ± 0.95 | 501 | 10 | 3.89 | 10.54 (5.64 - 19.70) | 2.08 (1.11 - 3.89) | 2.24 (1.19 - 4.21) | 28.92 ± 2.54 | 6950 | 126 | 3.49 | 9.40 (6.32 - 14.00) | 3.34 (2.24 - 4.98) | 3.45 (2.30 - 5.19) |
| 20 | 24.24 ± 0.82 | 196 | 6 | 6.10 | 16.57 (7.41 - 37.04) | 2.81 (1.26 - 6.30) | 2.94 (1.31 - 6.60) | 29.52 ± 2.72 | 5718 | 115 | 3.89 | 10.51 (7.03 - 15.70) | 3.49 (2.33 - 5.23) | 3.42 (2.27 - 5.18) |
|  | 22.11 ± 1.93 |  |  |  | p < 0.001 | p < 0.001 | p < 0.001 | 27.32± 2.09 |  |  |  | p < 0.001 | p < 0.001 | p < 0.001 |

Abbreviation: BMI, body mass index; AF, atrial fibrillation; IR, incidence rate; PY, person-years; HR, hazard ratio; CI, confidence interval.

Model 1 is unadjusted.

Model 2 is adjusted for age, sex, smoking status, alcohol intake, regular exercise, and low income.

Model 3 is adjusted for age, sex, smoking status, alcohol intake, regular exercise, low income, waist circumference, systolic blood pressure, fasting glucose, logarithm of TG, and HDL-C level.

Model 1 *P* for interaction = 0.1496

Model 2 *P* for interaction = 0.7608

Model 3 *P* for interaction = 0.7732

**Table S5**. The risk of atrial fibrillation after the first 2 years of follow-up according to the cumulative number of total MetS components diagnosed during four health examinations.

| The number of meeting individual components | No. of participants | AF | IR  (1000PY) | HR (95%CI) | | |
| --- | --- | --- | --- | --- | --- | --- |
|  |  |  |  | Model 1 | Model 2 | Model 3 |
| 0 | 278321 | 363 | 0.39 | 1.00 (Reference) | 1.00 (Reference) | 1.00 (Reference) |
| 1 | 297082 | 499 | 0.51 | 1.29 (1.13 - 1.48) | 1.07 (0.93 - 1.22) | 1.07 (0.93 - 1.23) |
| 2 | 282414 | 668 | 0.72 | 1.82 (1.60 - 2.07) | 1.31 (1.15 - 1.49) | 1.31 (1.16 - 1.50) |
| 3 | 264241 | 700 | 0.80 | 2.04 (1.80 - 2.32) | 1.30 (1.15 - 1.48) | 1.31 (1.15 - 1.49) |
| 4 | 262901 | 925 | 1.07 | 2.72 (2.41 - 3.07) | 1.51 (1.33 - 1.70) | 1.52 (1.34 - 1.72) |
| 5 | 234120 | 924 | 1.20 | 3.05 (2.70 - 3.45) | 1.55 (1.37 - 1.75) | 1.57 (1.38 - 1.78) |
| 6 | 209444 | 964 | 1.41 | 3.57 (3.17 - 4.03) | 1.68 (1.49 - 1.90) | 1.71 (1.50 - 1.94) |
| 7 | 186338 | 991 | 1.63 | 4.13 (3.67 - 4.66) | 1.84 (1.63 - 2.08) | 1.88 (1.65 - 2.13) |
| 8 | 172121 | 979 | 1.74 | 4.43 (3.93 - 4.99) | 1.86 (1.65 - 2.11) | 1.90 (1.67 - 2.16) |
| 9 | 143449 | 901 | 1.93 | 4.90 (4.34 - 5.54) | 2.00 (1.77 - 2.27) | 2.05 (1.80 - 2.34) |
| 10 | 121413 | 789 | 2.00 | 5.08 (4.49 - 5.75) | 2.00 (1.76 - 2.27) | 2.05 (1.79 - 2.34) |
| 11 | 100119 | 712 | 2.19 | 5.57 (4.91 - 6.32) | 2.13 (1.87 - 2.42) | 2.18 (1.90 - 2.50) |
| 12 | 88445 | 662 | 2.31 | 5.87 (5.16 - 6.67) | 2.15 (1.89 - 2.45) | 2.20 (1.91 - 2.54) |
| 13 | 67693 | 595 | 2.72 | 6.91 (6.07 - 7.88) | 2.47 (2.16 - 2.82) | 2.52 (2.18 - 2.91) |
| 14 | 51878 | 487 | 2.91 | 7.39 (6.45 - 8.46) | 2.56 (2.23 - 2.94) | 2.60 (2.24 - 3.02) |
| 15 | 38493 | 386 | 3.11 | 7.90 (6.84 - 9.11) | 2.69 (2.32 - 3.11) | 2.72 (2.32 - 3.19) |
| 16 | 32354 | 370 | 3.55 | 9.03 (7.81 - 10.44) | 2.90 (2.50 - 3.36) | 2.94 (2.50 - 3.45) |
| 17 | 17851 | 209 | 3.64 | 9.26 (7.81 - 10.98) | 2.94 (2.48 - 3.49) | 2.91 (2.42 - 3.50) |
| 18 | 11632 | 135 | 3.61 | 9.18 (7.53 - 11.18) | 2.88 (2.36 - 3.52) | 2.81 (2.27 - 3.47) |
| 19 | 7409 | 97 | 4.07 | 10.36 (8.28 - 12.96) | 3.24 (2.59 - 4.06) | 3.13 (2.47 - 3.97) |
| 20 | 5869 | 76 | 4.06 | 10.32 (8.06 - 13.22) | 3.07 (2.40 - 3.94) | 2.84 (2.19 - 3.69) |
|  |  |  |  | p < 0.001 | p < 0.001 | p < 0.001 |

Abbreviation: AF, atrial fibrillation; IR, incidence rate; PY, person-years; HR, hazard ratio; CI, confidence interval.

Model 1 is unadjusted.

Model 2 is adjusted for age, sex, smoking status, alcohol intake, regular exercise, and low income.

Model 3 is adjusted for age, sex, smoking status, alcohol intake, regular exercise, low income, waist circumference, systolic blood pressure, fasting glucose, logarithm of TG, and HDL-C level.
